# Wearable devices for remote monitoring of hospitalized patients with COVID-19 in Vietnam

**DOI:** 10.12688/wellcomeopenres.18026.2

**Published:** 2023-06-20

**Authors:** Nguyen Van Vinh Chau, Truong Ngoc Trung, Phan Nguyen Quoc Khanh Khanh, Phung Tran Huy Nhat, Hoang Minh Tu Van, Ho Bich Hai, Duong Bich Thuy, Nguyen Le Nhu Tung, Dao Bach Khoa, Tran Thi Dong Vien, Nguyen Van Hao, Pham Kieu Ngyuyet Oanh, Tran Dang Khoa, Nguyen Thanh Phong, Nguyen Thanh Nguyen, Julie Huynh, Timothy M Walker, Jennifer Van Nuil, Luu Phuoc An, Jacob McKnight, Le Mau Toan, Le Van Tan, Nguyen Thanh Dung, Nguyen Thanh Truong, C Louise Thwaites, OUCRU COVID Research Group

**Affiliations:** 1 Hospital for Tropical Diseases, Ho Chi Minh City, Vietnam; 2 Oxford University Clinical Research Unit, Ho Chi Minh City, Vietnam; 3 Kings College, London, UK; 4 University of Medicine and Pharmacy, Ho Chi Minh City, Vietnam; 5 Centre for Tropical Medicine and Global Health, University of Oxford, Oxford, UK; 6 Health Systems Collaborative, University of Oxford, Oxford, UK

**Keywords:** Wearable monitoring, Pulse oximetry, COVID-19, Low-middle-income countries, resource-limited

## Abstract

Patients with severe COVID-19 disease require monitoring with pulse oximetry as a minimal requirement. In many low- and middle- income countries, this has been challenging due to lack of staff and equipment. Wearable pulse oximeters potentially offer an attractive means to address this need, due to their low cost, battery operability and capacity for remote monitoring. Between July and October 2021, Ho Chi Minh City experienced its first major wave of SARS-CoV-2 infection, leading to an unprecedented demand for monitoring in hospitalized patients. We assess the feasibility of a continuous remote monitoring system for patients with COVID-19 under these circumstances as we implemented 2 different systems using wearable pulse oximeter devices in a stepwise manner across 4 departments.

## Introduction

Monitoring peripheral oxygen saturation (SpO _2_) is an essential component of management of moderate or severely ill patients with COVID-19, enabling timely institution of treatments such as oxygen or mechanical ventilation. Globally, the huge number of cases of COVID-19 has put health systems under enormous strain, and even simple monitoring tasks, such as SpO _2_ observations, are challenging due to lack of equipment and trained staff. In many low and middle income countries (LMICs) these were already known to be constraints in providing critical care and the Hospital for Tropical Diseases Ho Chi Minh City, collaborating with Oxford University Clinical Research Unit, were already developing innovative ways of improving real-time monitoring of critically ill patients in the intensive care unit (ICU) ^1^. Investigating the use of wearable monitoring systems formed part of this strategy as, in addition to lower cost, the wireless connectivity, small size and battery power offer considerable advantages for use in a resource-limited ICU where power supply and overcrowding are common issues ^1^. A specific multidisciplinary team had identified and tested wearable pulse oximeters (measuring SpO _2_) and electrocardiogram (ECG) devices for this purpose ^2^. Using these selected wearables, previous work had demonstrated that data collection is feasible and that data quality collected from these devices was comparable to data derived from state-of-the-art bedside ICU monitors, however a large-scale deployment of wearable monitoring devices had not been performed ^2^. Whilst particularly attractive in LMICs, the potential advantages of cost and automated data collection are also attractive in high income settings where wearable monitoring has been successfully used in post-surgical patients, and as a basis for early warning scores in ICUs ^3, 4^.

The huge demand for basic monitoring (particularly of SpO _2_) during the COVID-19 pandemic, led to several initiatives using wearable monitors in healthcare settings. The majority of those reported have been in high-income countries and in out-patient settings where patients’ self-reported intermittent values from wearable pulse oximeters have been used as means of triage or to facilitate early discharge of patients with relatively mild disease ^5– 7^. Successful automated monitoring of ambulatory patients with COVID-19 has been described using a system connecting wearable devices via bedside android tablets to clinician dashboards ^7^. Use of such systems in ICUs is more limited ^8, 9^. In high income settings, widespread availability of centralized bedside monitoring systems with wired-connectivity already provides staff with the means to remotely monitor patients’ vital signs continuously. However, in LMICs, where conventional pulse oximetry is often not available, simple low-cost systems have the potential for significantly improving patient management. Furthermore, implementation of pulse oximetry monitoring in resource-limited settings enables optimal Oxygen administration and has been shown to actually reduce overall Oxygen utilization ^10^. Ensuring a sustainable Oxygen supply for patients with COVID-19 continues to be a problem in many LMICs, and increasing pulse oximetry monitoring capacity is integral to achieving this ^11^.

**Figure 1.**
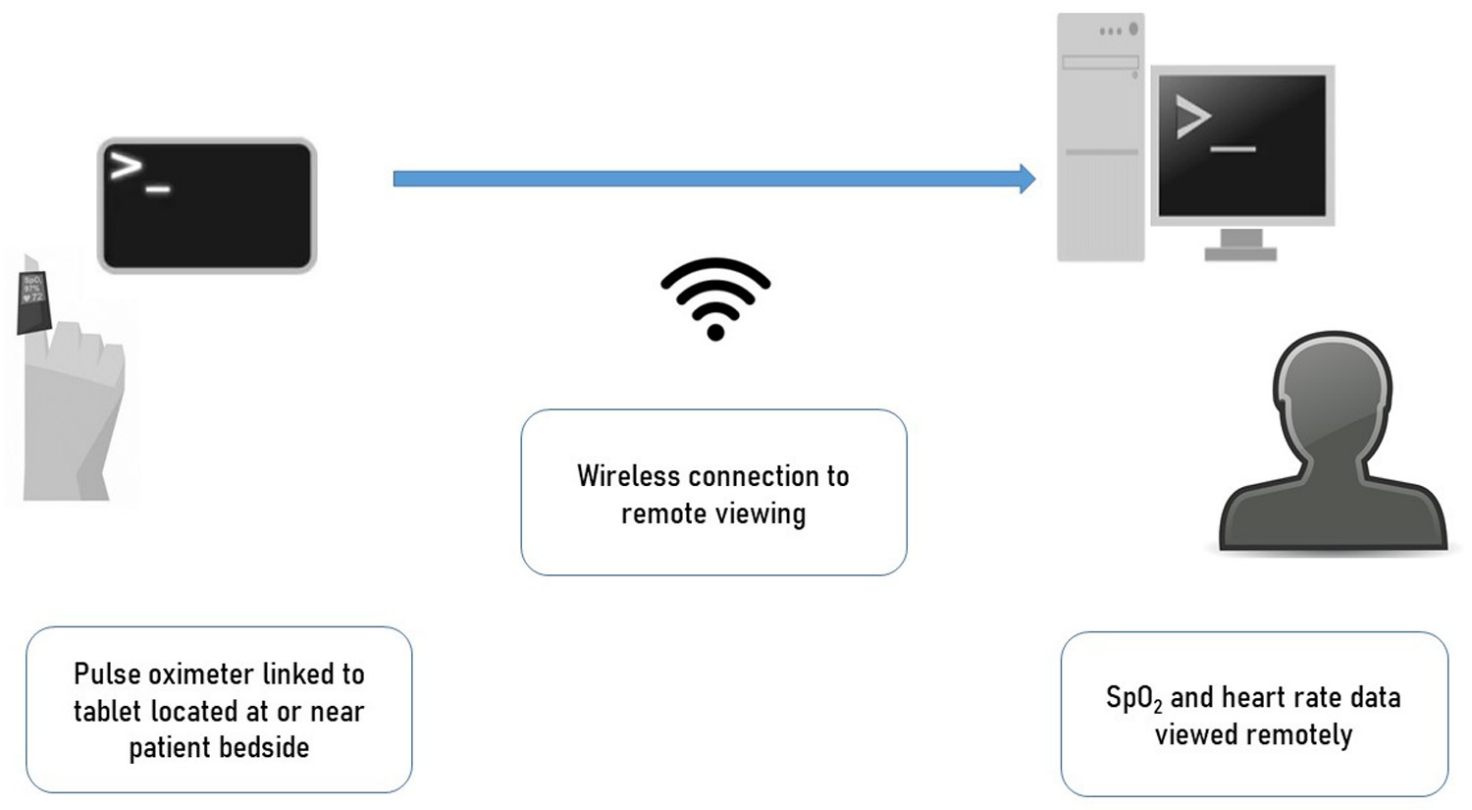
Schematic representation of the monitoring systems used. Wearable pulse oximeters were connected with tablet/phones placed either by the bedside or nearby (outside room). These connected through either screen mirroring applications or direct wireless connections to enable remote viewing by medical staff in central staff workstations.

At the outset of the pandemic a wearable pulse oximeter system was piloted in a field-hospital setting in Vietnam, using a screen sharing mobile application to access a bedside mobile device connected to a wearable pulse oximeter ^12^ ( Figure 1). Patients using this system were mobile and able to use this themselves (using the telephone for additional support). Following a surge in cases in Ho Chi Minh City June-August 2021, our team responded to a local need for expansion of monitoring capacity by implementing this in more severely-ill patients in high-dependency/ICU wards at the Hospital for Tropical Diseases. A second wearable-based pulse oximetry monitoring system was also implemented. This was similar in that it utilized a wearable pulse oximeter connected to a Bluetooth tablet but contained a bespoke user-interface and clinician dashboard capable of simultaneously displaying data from multiple patients. Both systems were capable of providing continuous SpO _2 _and heart rate measurements, accessible both at the bedside and remotely. 

Here we describe the implementation process at the Hospital for Tropical Diseases, a large infectious disease hospital, during the COVID-19 pandemic, highlighting the key challenges that the COVID situation presented and potential benefits for future care of ICU patients in LMICs.

## Methods

The Hospital for Tropical Diseases is am 800-bed tertiary referral hospital for infectious diseases, serving southern Vietnam (population 40 million). The Oxford University Clinical Research Unit has been hosted within the hospital for almost 30 years, aiming to work closely with HTD improving outcomes from patients with infectious diseases. At the outset of the pandemic (January 2021) a small number of isolation rooms were used to treat patients with COVID-19, however as the pandemic progressed increasing numbers of departments were given over to treating COVD-19 patients. By July 2021, full ICU facilities were available in 6 departments (4 of which were included in this project). A multidisciplinary team was involved in the project from the outset and was comprised of clinicians, managers, intensive care specialists, data scientists, industry experts and biomedical engineers. Health systems scientists were subsequently incorporated into the team. At the outset of the project clear responsibilities of each party were defined, including device management, data governance and ownership. Key hospital stakeholders defined the basic requirement for monitoring systems: namely the ability to continuously visualise individual patients’ SpO _2_ remotely. Routine vital sign monitoring (intermittent manual measurements by bedside nursing staff) were measured and recorded in the hospital electronic and paper records as normal.

Face-to-face formal meetings involving all parties were often impossible due to social-distancing restrictions. A central communication system through one or two key individuals was established and electronic communication though email or encrypted social media groups was used to keep regular updates. An online ‘problem’ sheet was instituted at an early phase to allow issues to be communicated rapidly. Many of the workflows occurred in parallel due to the nature of the work.

The majority of the work focussed on institution of the new system (System A), as this had capability for monitoring more patients with access to a larger number of available compatible monitoring devices, at a time when monitoring devices were in short supply. Implementation and ongoing development of this system was also supported by an industry team. For example, the system was able to incorporate additional features specified by frontline workers such as a ‘traffic-light alarm’ system and adapt the clinician dashboard to staff requirements. Based on these user requirements defined by hospital stakeholders, a minimally viable product was created for System A ( Figure 1). This consisted of wearable pulse oximeters (Viatom Viatomtech, China) connected to a tablet in a 1-to-1 routine configuration, connected to a cloud server via 3G network allowing data visualization from all monitors on a computer screen. This system was piloted off-site in healthy people first then introduced into the ward environment where a further pilot was held using devices in a non-patient area. Following this, the prototype system was introduced into one department in a small number of patients following staff training.

In addition to this new system, the previously-piloted adaptive remote monitoring system (System B) was introduced in one department. The system, previously described, consisted of a wearable pulse oximeter (SmartCare, SmartCare Analytics, UK) connected to an android tablet via a Bluetooth connection (1-to-1 configuration). The pulse oximetry readings from several devices could be viewed in real-time on a remote computer screen via a screen mirror application which allowed mirroring of multiple devices, although not simultaneously. In addition to this method, ward staff could also use the departments’ close circuit television (CCTV) systems’ ‘zoom in’ function to view the bedside tablet’s display of pulse oximetry output. Thus, although both System A and B were able to display continuous heart rate and pulse oximetry, multiple patient data could be viewed using System A which also included additional alert data for abnormal values. System B however allowed detail waveform pulse oximetry data to be viewed and bedside tablets served as additional bedside monitoring displays which could be viewed by CCTV camera.

As ward staff were under extreme pressure, responding ‘ad hoc’ to technical issues such as battery run-out was not feasible, thus for both systems, each patient was allocated two wearable devices to allow constant monitoring (one device active and one being charged). These devices were changed at fixed times in the day irrespective of battery levels. Standard infection-control procedures were employed to clean devices between patients. Research team members were also directly involved in the implementation, supporting these activities. In addition, routine vital sign observations procedures were carried out as normal by clinical staff following hospital and Vietnamese Ministry of Health guidelines.

## Results

Between August 3 ^rd^ and October 31 ^st^ 2021, the two monitoring systems were implemented in four dedicated departments for patients with COVID-19 at the Hospital for Tropical Diseases. These departments mainly received patients with severe and critical COVID-19 according to Vietnamese Ministry of Health Guidelines ^13^, and all had ICU facilities for additional respiratory support (facemask oxygen, nasal oxygen, high flow nasal oxygen, non-invasive ventilation and invasive mechanical ventilation). Table 1 shows the total number of patients admitted to the four departments in which wearable monitoring was instituted between August and October 2021.

**Table 1.** Showing number of patients admitted to departments involved in the project August–October 2021.

Month	Ward A	Ward D	Ward E	A-ICU
**August**	104	126	189	37
**September**	72	83	107	33
**October**	22	75	94	13
**Total**	198	284	390	83

**Figure 2.**
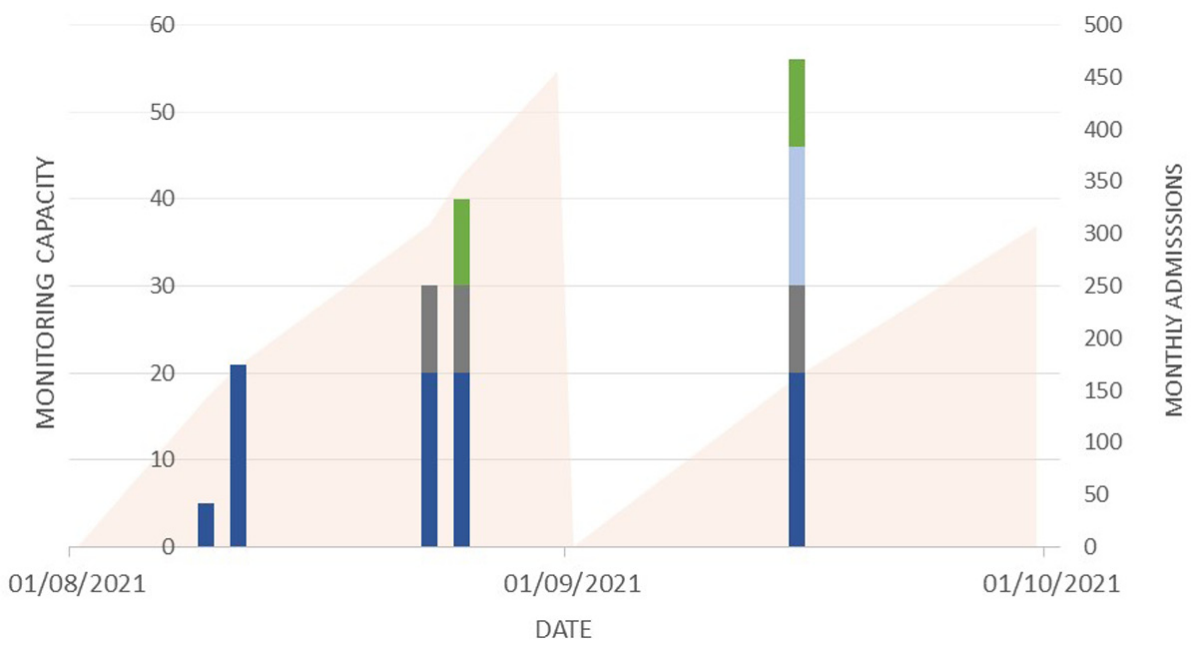
Figure showing stepwise scale up of wearable monitoring within hospital departments (Stacked bars). System A was introduced in Departments E (dark blue), AICU (grey) and A (light blue) with system B in Department D (green). Solid area represents cumulative new admissions to these 4 departments during the month.

The new system (System A) was instituted in three of the departments and in one (Ward D), the previously-piloted (System B) was implemented. The phased scale up of this is shown in Figure 2 and the monthly cumulative admissions to the four departments over the same time period. 

The initial challenges encountered were practical issues setting up the basic infrastructure required to deploy the wearable systems, given the extreme clinical pressures at the time and limited availability of new equipment. During the set-up phase, the hospital completed infrastructure upgrades in several of the departments where the devices were deployed, for example ensuring oxygen supply, electric supply for equipment and physical infection control barriers. In parallel with this, any necessary hardware was installed, repurposing equipment such as monitors and laptop computers to reduce the need to purchase new equipment as much as possible. 

System A was the first system introduced, with the first patients monitored within four weeks from the project outset. An initial pilot of five sets (each two devices), able to monitor five patients was scaled up to increase monitoring capacity for 20 patients within Ward E. The research team worked closely with the clinical team and the industry partner to troubleshoot technical problems as they arose. Following this phase, the system was introduced into two other departments: (Ward AICU and Ward A). System B was implemented in Ward D and provided capacity for 16 patients, with bedside tablets connected to a laptop allowing staff to remotely view monitoring data from outside the patient area. 

We observed that the exact use of the wearable systems varied according to the ward. In the Wards A, D and E, the systems provided continuous monitoring in for individual patients who would otherwise receive intermittent manual measurements. In addition to using the clinician dashboard (System A), or laptop (System B), bedside tablets also served as equivalent of bedside monitors, enabling staff at the bedside to see continuous vital sign data. In AICU, where conventional bedside monitoring was already available, the wearables were used in patients in whom no remote-viewing facility was available, thus patients’ SpO _2_ data could also be monitored from a central station outside of the patient areas.

## Discussion

We have described the feasibility of implementing a wearable monitoring system in our hospital in a situation of exceptional healthcare system stress in Ho Chi Minh City, where there was a constant requirement for the system to respond to the evolving pandemic. Under these circumstances, our main aim was to rapidly set up a functioning system, that could complement current standard of care, using expertise and equipment available at the time. The urgent need for increased monitoring capacity meant that a ‘lean’ implementation approach was required – i.e. systems functioning with minimal requirements were introduced first and adapted over time. This was a big advantage in that it enabled rapid deployment and additional level of remote monitoring when there was a large unmet need. Our first wearable devices were deployed in patients in less than four weeks, from first conceptualization. Undoubtedly our teams’ previous experience and multidisciplinary expertise were enabling factors in achieving this, despite the new challenges of operating in COVID-19 context ^14^.

 As expected, challenges arose due to the novelty of the technology and speed at which it needed to be implemented. Our pre-pandemic experience, while useful for this, did not directly translate for the pandemic use. For example, support and technical staff were no longer easily able to access patients’ bedsides (or required full PPE to do so), and staff previously familiar with the devices had been located to other departments.

Similar to other LMICs, even outside of the pandemic, staff-to-patient ratios in Vietnamese hospitals are significantly lower than generally reported in high-income settings ^15^. Accordingly, staff availability for training in operation and trouble-shooting of new devices may be limited. In the pandemic context, the constantly-changing capacity and structure of the city’s healthcare system and the implementation of specific care pathways necessitated frequent staff redeployment into and out of the departments described herein. As a consequence, additional time was required to train new staff in the systems, which under normal circumstances would have been less. The presence of dedicated research staff to support the project we believe was vital in enabling this ongoing training and education. Notably, however many of these staff had been redeployed from other research projects.

Although the features of battery power and blue tooth connectivity were attractive features for our immediate need, both were also aspects of the system requiring specific workflows to charge devices and maintain connectivity. In the UK, a similar wearable monitoring system was implemented in a high-dependency setting in a total of 59 patients between March and August 2020. Notably, in this context, there were significantly more staff available (Four to five nursing staff and three to four doctors for 19 patients) ^7^. Staff responded to problems as they arose however in our setting, regular ‘maintenance’ schedules were found to be more effective ways of addressing these issues with fewer staff available and in view of the rapid turnover.

A limitation of this work is the lack of detailed operational data to more thoroughly describe the function of the deployed systems, for example of patient monitoring, and number of devices used at any one time. In the UK example above, the system was already developed prior to the pandemic and data metrics easily taken from this. As pandemic pressures ease, incorporating these metrics is an important step – both technically and clinically. Similarly, usability feedback from staff is also now an important consideration, but at the time, formal evaluation was limited by staff availability to both give and record feedback.

## Conclusion and next steps

Our project shows how important pre-existing expertise and infrastructure were in rapidly implementing our solution. As we, hopefully, face a fading pandemic, the fundamental issues of providing high-quality low-cost critical care in LMICs remain, and central to this is the need to provide low-cost clinical monitoring. We aim to direct the learning and expertise gained in this project towards achieving this. Whilst our efforts in this project were directed at a specific pandemic-related solution, notably absent was capture and analysis of monitoring data. Recent advances in analytics (particularly artificial intelligence/ machine learning) offer exciting potential for decision support systems based on such data, and could be used to support patient care and improve patient outcomes in LMICs. Designing and implementing an effective monitoring solution with such capacity would have immense potential for care of critically ill patients in low-resource settings.

## Data Availability

Data included in this manuscript are provided by the Hospital for Tropical Diseases and are available through OUCRU and HTD data sharing processes.
