# Supplementary material for: Wearable devices for remote monitoring of hospitalized patients with COVID-19 in Vietnam
Source: Wellcome Open Res. 2023 Jun 20;7:257. Originally published 2022 Oct 12. [Version 2] doi: 10.12688/wellcomeopenres.18026.2 (PMC11004598; doi:10.12688/wellcomeopenres.18026.2)
Supplement: Peer review history

# Reviewer report: Reviewer response for version 2

**DOI:** 10.21956/wellcomeopenres.21655.r70228

**Published:** 2024-04-13

**Authors:** Jonathan Clarke

**Affiliations:** 1 Centre for Mathematics of Precision Healthcare, Imperial College London, London, UK

Thank you for giving me the opportunity to review this article describing the implementation of two pathways to monitor patients with Covid-19 in hospitals in Vietnam using digital technologies. The article is well written overall, with a few typos identified and described below.

 The authors acknowledge the limitations of the study in terms of their ability to evaluate the usability of the pathway or the patient benefit obtained from its implementation. As such, the article focusses instead on describing an intervention which may be of use to other health systems, or for which important lessons could be learned. With this in mind, I wonder if some of the discussion could be given over to clearly describing what the authors have learned from this experience that may be of use to others in a similar position. This set of recommendations based on their experience of a real-world rollout of a new technology at pace and amidst significant constraints are likely to be useful and of interest to a wider audience.

 Several minor points for clarification in order to enable replication of the work or clear interpretation of the findings are described below.

 Minor points:

 Introduction:

 - Please clarify that ‘Android’ refers to the operating system of the tablets

 - Clarify what a ‘screen sharing mobile application’ is

 Consider ensuring consistency in capitalisation of ‘oxygen’ across the article.

 - Re: ‘patients using this system were mobile’ - were they inpatients, or was this part of a remote / home monitoring programme?

 - It may be useful to provide a little context re: Vietnam’s COVID-19 timeline / policies - i.e. its zero-Covid policies avoided significant numbers of cases until April 2021. I can imagine some readers getting confused around timelines as they differ from other countries.

 Methods:

 - Define HTD on first use

 - Typo - ‘am’ / ‘an’

 - As described in the methods - System A aggregated multiple patient readings through simultaneously receiving the screens of several separate tablets, rather than parsing the clinical data and then representing it in a different format?

 - I agree with the suggestion of the previous reviewer to include System B in Figure 1 to clarify the differences between it and System A.

 - What did the piloting on healthy individuals and a non-patient area involve? What did it aim to achieve, what was learned?

 Results:

 - Typo ‘continuous monitoring for individual patients’ rather than ‘continuous monitoring in for individual patients’

 - Figure 2: Why was cumulative monthly admissions chosen to represent activity over the study period? Would either daily or weekly admissions or cumulative over the entire period be more useful? Daily or weekly admissions may give a clearer indication of changing activity over time than interpreting the gradient of a cumulative plot that resets half way through. The vertical bars represent the capacity of a unit at the time when its capacity changes. This is quite hard to interpret and would perhaps be better visualised as a line for each unit showing its capacity over time. This would allow ready comparison and avoid confusion about the capacity of a unit between the bars.

Is the rationale for developing the new method (or application) clearly explained?

Yes

Is the description of the method technically sound?

Yes

Are the conclusions about the method and its performance adequately supported by the findings presented in the article?

Yes

If any results are presented, are all the source data underlying the results available to ensure full reproducibility?

No source data required

Are sufficient details provided to allow replication of the method development and its use by others?

Partly

Reviewer Expertise:

Health policy, Health services research, Digital health, Public health

I confirm that I have read this submission and believe that I have an appropriate level of expertise to confirm that it is of an acceptable scientific standard.

# Reviewer report: Reviewer response for version 2

**DOI:** 10.21956/wellcomeopenres.21655.r64776

**Published:** 2024-04-09

**Authors:** Deepak Berwal

**Affiliations:** 1 Florida Atlantic University, Boca Raton, Florida, USA

The abstract provides a clear overview of the study, outlining the need for wearable pulse oximeters in monitoring COVID-19 patients in low-resource settings. It effectively summarizes the study's objectives and methodology. However, it lacks specific findings or results. Adding a sentence about the key findings would enhance its completeness.

 The methods section provides a detailed description of the study's setting, the two monitoring systems used, and the challenges faced during implementation. However, it lacks specific technical details about the wearable devices, which could be valuable for readers interested in replicating the study. Including information about the make and model of the devices, their technical specifications, and how they were integrated into the healthcare workflow would enhance this section.

 There are a couple of articles like [1] and Analysis of Reflectance Photoplethysmograph Sensors (zenodo.org). Authors can consider multiple factors like contact pressure, temperature, motion artifacts etc. to report the accuracy of the outcomes and compare the wearable device with already existing standard devices. The following two articles can be taken into consideration while doing this.

 The results section provides a clear timeline of when the monitoring systems were implemented and in which departments. However, it lacks specific data on patient outcomes or the performance of the monitoring systems. Including information on the number of patients monitored, key findings, and any issues encountered during the monitoring period would make this section more informative.

 The discussion section effectively summarizes the challenges faced during the implementation of the monitoring systems and the importance of pre-existing expertise and infrastructure. It also touches on the limitations of the study. However, it could benefit from a more in-depth discussion of the potential implications of this work, such as the scalability of wearable monitoring systems in LMICs and the need for data analytics for decision support.

**Recommendation**: I recommend revising the manuscript to include specific data on patient outcomes, the performance of the monitoring systems, and technical details about the wearable devices used. Additionally, consider discussing the broader implications of this work and its potential for scalability and data-driven decision support in LMICs.

Is the rationale for developing the new method (or application) clearly explained?

Partly

Is the description of the method technically sound?

Partly

Are the conclusions about the method and its performance adequately supported by the findings presented in the article?

Partly

If any results are presented, are all the source data underlying the results available to ensure full reproducibility?

Yes

Are sufficient details provided to allow replication of the method development and its use by others?

No

Reviewer Expertise:

Wearable devices, Healthcare systems, and Biomedical signals

I confirm that I have read this submission and believe that I have an appropriate level of expertise to confirm that it is of an acceptable scientific standard, however I have significant reservations, as outlined above.

## References

[1] : SpO2 Measurement: Non-Idealities and Ways to Improve Estimation Accuracy in Wearable Pulse Oximeters. *IEEE Sensors Journal* .2022;22(12) : 10.1109/JSEN.2022.3170069 11653-11664 10.1109/JSEN.2022.3170069

# Reviewer report: Reviewer response for version 2

**DOI:** 10.21956/wellcomeopenres.21655.r75888

**Published:** 2024-04-09

**Authors:** Benjamin Smarr

**Affiliations:** 1 Department of Bioengineering, University of California San Diego Health System, San Diego, California, USA

This paper identifies limitations of wired infrastructure for monitoring patients during times of surging hospital load, as in COVID-19 peaks of infection. Based on this, they test the feasibility of using wireless devices to augment hospital monitoring capacity, using a single hospital in Ho Chi Minh City. I have very little to add as this is a well framed report.

 This paper seeks to address specifically the use of wireless O2 monitors instead of wired monitors within a hospital setting. The paper is clearly written, though the introduction could be reduced if needed given the scope. It has a limited scope of assessing staff's ability to interact with wireless O2 sensor solutions, but it makes clear that it addresses the scope it sets. The language is easy to read and the Methods are interesting and may help others adopt these practices.

Is the rationale for developing the new method (or application) clearly explained?

Yes

Is the description of the method technically sound?

Yes

Are the conclusions about the method and its performance adequately supported by the findings presented in the article?

Yes

If any results are presented, are all the source data underlying the results available to ensure full reproducibility?

Yes

Are sufficient details provided to allow replication of the method development and its use by others?

Yes

Reviewer Expertise:

this field says "optional" but flags as required.

I confirm that I have read this submission and believe that I have an appropriate level of expertise to confirm that it is of an acceptable scientific standard.

# Reviewer report: Reviewer response for version 1

**DOI:** 10.21956/wellcomeopenres.19987.r56596

**Published:** 2023-05-16

**Authors:** Tamara Vagg

**Affiliations:** 1 Department of Computer Science and Medicine, University College Cork, Cork, County Cork, Ireland

Thank you for the opportunity to review this manuscript. It is very well written (with the exception of one or two typos) and is a very interesting example of the practical application of digital health. The manuscript describes two systems that use a wearable device coupled with a visualisation platform to view continuous SpO2 and heart rate data in multiple wards (including ICU) during the COVID-19 pandemic. While I agree with the authors limitations, that some sort of metrics to support the feasibility of the systems would further contribute to this work – I feel that a strength of this work is the detail of the real-world deployment and integration which was driven by the necessity of the pandemic. As it is currently written, enough detail has been given so that it can be reproduced by others. And I hope that the authors would consider the flow diagram outlined in “Optional Suggestion”.

***Minor Suggestions:***

**Figure 1: **Would it be possible to expand to describe the nuanced differences between the two monitoring systems(A+B)?

**Table 1: **Would it be possible to expand Table to include which monitoring system was being used in each ward?

***Optional Suggestion:***

 The stages of this system setup and deployment are very interesting. If it was allowed and agreeable by the authors, it would be fantastic to have some sort of flow diagram to describe the various phases (and paralleled workflows) and perhaps the different teams involved *e.g* ideation -> testing in field hospital -> infrastructure establishment -> pilot system x with ward x (n=x) -> further roll out *etc.*

Is the rationale for developing the new method (or application) clearly explained?

Yes

Is the description of the method technically sound?

Yes

Are the conclusions about the method and its performance adequately supported by the findings presented in the article?

Yes

If any results are presented, are all the source data underlying the results available to ensure full reproducibility?

Yes

Are sufficient details provided to allow replication of the method development and its use by others?

Yes

Reviewer Expertise:

Digital Health, Virtual Reality, Telehealth, Virtual Monitoring, mHealth, eHealth, Design, CF

I confirm that I have read this submission and believe that I have an appropriate level of expertise to confirm that it is of an acceptable scientific standard.

# Response

**Published:** 2023-06-03

**Authors:** C Louise Thwaites

**Affiliations:** Oxford University Clinical Research Unit, Vietnam

Thank you for these comments. We will include these suggestions into a further version of this article.
